# NMD and microRNA expression profiling of the *HPCX1 *locus reveal *MAGEC1 *as a candidate prostate cancer predisposition gene

**DOI:** 10.1186/1471-2407-11-327

**Published:** 2011-08-02

**Authors:** Henna Mattila, Martin Schindler, Jarkko Isotalo, Tarja Ikonen, Mauno Vihinen, Hannu Oja, Teuvo LJ Tammela, Tiina Wahlfors, Johanna Schleutker

**Affiliations:** 1 Institute of Biomedical Technology, University of Tampere and Centre for Laboratory Medicine, Tampere University Hospital, Tampere, Finland; 2 Bioinformatics, Institute of Biomedical Technology, University of Tampere and Science Center of Pirkanmaa Hospital District, Tampere, Finland; 3 School of Health Sciences, University of Tampere, Tampere, Finland; 4 Department of Urology and Medical School, Tampere University Hospital and University of Tampere, Tampere, Finland

## Abstract

**Background:**

Several predisposition loci for hereditary prostate cancer (HPC) have been suggested, including *HPCX1 *at Xq27-q28, but due to the complex structure of the region, the susceptibility gene has not yet been identified.

**Methods:**

In this study, nonsense-mediated mRNA decay (NMD) inhibition was used for the discovery of truncating mutations. Six prostate cancer (PC) patients and their healthy brothers were selected from a group of *HPCX1*-linked families. Expression analyses were done using Agilent 44 K oligoarrays, and selected genes were screened for mutations by direct sequencing. In addition, microRNA expression levels in the lymphoblastic cells were analyzed to trace variants that might alter miRNA expression and explain partly an inherited genetic predisposion to PC.

**Results:**

Seventeen genes were selected for resequencing based on the NMD array, but no truncating mutations were found. The most interesting variant was *MAGEC1 *p.Met1?. An association was seen between the variant and unselected PC (OR = 2.35, 95% CI = 1.10-5.02) and HPC (OR = 3.38, 95% CI = 1.10-10.40). miRNA analysis revealed altogether 29 miRNAs with altered expression between the PC cases and controls. miRNA target analysis revealed that 12 of them also had possible target sites in the *MAGEC1 *gene. These miRNAs were selected for validation process including four miRNAs located in the X chromosome. The expressions of 14 miRNAs were validated in families that contributed to the significant signal differences in Agilent arrays.

**Conclusions:**

Further functional studies are needed to fully understand the possible contribution of these miRNAs and *MAGEC1 *start codon variant to PC.

## Background

Prostate cancer is the most common form of cancer affecting men in the Western world. In Finland, there were 4234 new cancer cases diagnosed in 2008, and the incidence of prostate cancer (PC) was 82.9/100.000 [1]. In addition to age, a well-established risk factor for PC is a family history of the disease. In a large Scandinavian twin study [2], it was reported that approximately 40% of the risk for PC can be explained by heritable components. This proportion is the highest ever reported for a common malignancy. Most of the genes that are involved in the causation of hereditary cancers have been identified by linkage analysis. Several linkage studies of hereditary prostate cancer (HPC) have been performed and the results have implicated many risk loci located on different chromosomes, which indicates a great heterogeneity of this disease [3]. One of the loci found by linkage analysis is *HPCX1 *(OMIM %300147), which is located on chromosome Xq27-q28 [4]. This locus has proven to be important in the Finnish population [5] and the region around the best linkage marker was found to be in strong linkage equilibrium [6]. However, the susceptibility gene has not yet been identified because the chromosomal region has an extremely complex genomic structure with multiple gene duplications and inversions that have hampered conventional gene cloning methods [7]. The *SPANX *genes and *LDOC1 *at Xq27 have been considered to be the best positional candidate genes for *HPCX1*, but no direct evidence for causative mutations in any of the genes studied have been detected [8,9].

Mutations, especially nonsense mutations, in tumor suppressor genes (TSGs) are common in the development and progression of cancer. They give rise to in-frame premature translation termination codons within the coding regions of genes and lead to truncated protein translation products. However, the identification of TSGs by classical cancer genetics methods is difficult and slow. In addition, RNA transcripts carrying nonsense mutations are usually targeted for degradation through nonsense-mediated decay (NMD) [10]. NMD is a complex process in mammalian mRNA metabolism, and its function is to eliminate faulty transcripts and control the expression of normal genes.

A conventional strategy for the identification of disease genes is to use microarrays to compare the levels of gene-specific mRNA expression between patient and control samples. However, identification of the mutated gene can be obscured by inter-individual variation and secondary changes in gene expression caused by the disease process. Noensie and Dietz [11] reported an alternative strategy that circumvents these limitations, called GINI (Gene Identification by NMD Inhibition), in which the patient sample is compared to itself after the pharmacological inhibition of NMD. Microarrays are then used to identify potential nonsense transcripts that are increased in abundance after the loss of NMD. Emetine was used to block the pathway, but was problematic as emetine induces a stress response that results in the upregulation of additional transcripts. Ionov et al. [12] combined the emetine treatment with actinomycin D, which effectively prevents the upregulation of stress response genes while still stabilizing mutant transcripts.

Inactivation of autosomal tumor suppressor genes is a two-step process involving the mutation of the target gene and the loss of the wild type allele. In lymphoblastoid cell lines established from patient samples, the normal wild type allele can mask the effect of a mutated allele. However, because males have only one X chromosome, there is only one allele of the X chromosomal germline genes. Therefore, truncated tumor suppressor mRNAs may be identified by using NMD method applied for RNA extracted from patients' lymphoblastoid cell lines.

Cancer is fundamentally a disease of disordered gene expression. Since no causative mutations have been identified from the transcribed genes of the *HPCX1 *region, it is possible that the defect occurs at the regulatory level. Regulatory defects might also explain the relatively late onset of the disease, assuming a polygenic model for PC development with additive effect to the phenotype. MicroRNAs (miRNAs) are small RNA molecules that regulate gene expression post-transcriptionally [13]. They play an important role in diverse biological processes and, accordingly, altered miRNA expression is likely to contribute to human disease, including cancer. It has been shown that miRNA profiles are surprisingly informative. They become altered with the development and progression of PC [14] and have a very important role in the biology of the disease [15]. Since considerable amount of miRNAs are located within intronic regions and regulated by the host gene promoter [16], miRNA expression profiles give us one possibility to study disease related variations in non-protein coding chromosomal areas and this could lead to identification of regulatory variants especially in region with complex genomic structure like one in chromosome X.

Here, we present a study with Finnish multiplex *HPCX1 *linked families in which we have characterized the *HPCX1 *locus by NMD and miRNA microarray methods and evaluated the role of *HPCX1 *in the causation of familial prostate cancer.

## Methods

### Study population

#### NMD microarray analysis

Collection of the Finnish families with PC has been reported previously [5]. Based on the first linkage to *HPCX1 *in Finland [5], six affected and six healthy males for controls were selected from the linked families for NMD microarray analysis. Controls were the oldest healthy brothers of the affected males. The clinical characteristics of the patients are described in Table 1.

**Table 1:** Demographic, clinical, and pathological characteristics of the patients in microarray analyses

Patient	Diagnosis age	Gleason score	WHO grade	T*	**N **^†^	**M **^‡^	**Primary PSA**^§^
015-001**	53 y 7 m	n.a.	II	T1c	Nx	M0	n.a.
043-001**	50 y 9 m	6	II	T2	Nx	M0	3.8
232-001**	70 y 7 m	7	II	T1c	Nx	M0	6.2
232-002**	75 y 5 m	n.a.	n.a.	T1a	Nx	M0	1.3
248-006**	59 y 3 m	6	I	T1c	Nx	M0	2.9
311-003**	60 y 4 m	6	II	T2	Nx	M0	21.9
001-002	71 y 8 m	n.a.	II	T3	Nx	M0	n.a.
292-010	47 y 11 m	6	II	T2	Nx	Mx	5.2
408-002	67 y 5 m	7	II	T1c	Nx	Mx	5.5
236-006	75 y 11 m	n.a.	n.a.	T3	Nx	Mx	85
402-003	67 y 3 m	6	I	T2	Nx	M0	13
402-001	67 y 11 m	7	II	T1c	Nx	M0	15
413-003	55 y 3 m	7	II	T1c	Nx	M0	6.4
362-001	50 y 7 m	6	I	T1b	Nx	M0	6.9
362-002	49 y 4 m	6	I	T1c	Nx	Mx	41.9

*The size of the tumor

^†^Regional lymph nodes

^‡^Distant metastases

^§^Prostate specific antigen

**Patients in the NMD array

#### miRNA microarray analysis

For the miRNA microarray analysis, the original number of *HPCX1*-linked families was increased by seven based on recent linkage analysis [17]. As in the NMD study, the oldest possible healthy brother of the affected males was chosen as the control. The clinical characteristics of the patients are described in Table 1.

#### Association analysis

The P.Met1? variant was analyzed in the youngest affected patient from 163 HPC families, 757 patients with unselected PC, 757 healthy male blood donors, 764 healthy female blood donors, 375 men with benign prostate hyperplasia (BPH), and 746 men who had a PSA level of less than 1.0 ng/ml (PSA controls). Collection of the Finnish families with PC has been reported previously [5]. In brief, families used in the association study had two or more first- or second-degree affected relatives. The mean number of affected relatives was 2.8 (range 2-7) and the mean age at diagnosis for the probands was 63.0 years (range 43-86). The youngest affected individual from each family was initially used in the association analysis. The unselected cases included consecutive patients diagnosed with PC in the Pirkanmaa Hospital District from 1999-2001. The mean age at diagnosis of the men with unselected PC was 69.0 years (range 45-93). The men with BPH were also patients from the Pirkanmaa Hospital District. The diagnosis of BPH was based on lower urinary tract symptoms, free uroflowmetry, and evidenced by palpation or transrectal ultrasound of increased prostate size. If PSA was elevated, then the patients underwent biopsies to exclude PC. The indication for biopsy was a total PSA level of ≥ 4 ng/ml or a total PSA level of 3.0-3.9 ng/ml with the proportion of free PSA < 16%. The mean age of the BPH patients was 73 years. The PSA controls were from the Finnish population-based prostate cancer screening trial [18]. The mean age of the men was 67.5 years (range 64-74). The population controls consisted of DNA samples from anonymous male and female blood donors obtained from the Finnish Red Cross in Tampere.

Permission to collect and use blood samples and clinical data from prostate cancer patients was granted by the Institutional Review Board of Tampere University Hospital and City of Tampere. Written informed consent for use of their samples as well as medical records was obtained from all individuals participating in this study.

### Cell culture and drug treatments

In the NMD microarray experiment, the cell lines were derived by Epstein-Barr virus transformation of peripheral mononuclear leukocytes from patients and their healthy brothers. Lymphoblastoid cell lines were grown in RPMI-1640 medium (Lonza, Walkersville, MD, USA) supplemented with 10% fetal bovine serum (Sigma-Aldrich, St. Louis, MO, USA) and antibiotics. The emetine treatment protocol was described previously [12]. Briefly, for each cell line, we treated half of the subconfluent cells with 100 μg/ml of emetine dihydrochloride hydrate (Fluka, Buchs, Switzerland) and the rest were used as untreated controls. Both the treated and untreated cells were incubated for 10 h at 37°C. After this, actinomycin D (Sigma-Aldrich) was added to the treated and untreated cells, and they were incubated for 4 h at 37°C. Cell pellets were snap-frozen and total RNA was extracted from treated and untreated cells with Trizol according to the manufacturer's instructions (Invitrogen, Carlsbad, CA, USA). In the miRNA microarray experiment, the lymphoblastoid cell lines were grown similarly as above, but without drug treatments. RNA yields were quantified using an ND-1000 spectrophotometer (Nanodrop Technologies, Wilmington, DE, USA).

### Oligonucleotide array protocol

mRNA levels in the treated and untreated cells were measured using the Agilent 44 K array according to the manufacturer's instructions (Agilent Technologies, Inc., Santa Clara, CA, USA). Twenty micrograms of total RNA were used to generate fluorescent Cy-3-labeled cRNA (control cells) and Cy-5-labeled cRNA (treated cells) using an Agilent Fluorescent Direct Label Kit. Labeled RNAs were pooled and hybridized to the Agilent 44 K Whole Human Genome Oligonucleotide Microarrays (Agilent Technologies) containing over 33,000 known and novel human genes (~41,000 human genes and transcripts). A total of 12 arrays were hybridized, one for every individual. Microarray slides were scanned (Agilent microarray scanner) after hybridization, and data was extracted using Feature Extraction software, version A.7.5.1. (Agilent Technologies). For data analysis, the raw microarray expression values of the rMeanSignal and gMeanSignal variables were first background-adjusted and a natural logarithm of a ratio of the variables rMeanSignal and gMeanSignal was taken. The log-ratio values were normalized between arrays by using the quantile normalization method. A linear mixed model was then used as a method for identifying the set of differentially expressed genes in a considered experimental set-up. The normalized expression values of each gene at Xq27-28 were separately modeled by the linear mixed model that included the treatment effect (i.e., the affected person versus healthy) as a fixed effect and the family effect as a random effect. In the model analysis, a considered gene was declared differentially expressed if the calculated estimate for the parameter associated with the fixed treatment effect was greater than zero, and if, at the same time, the p-value in the t-test for the null hypothesis concerning the fixed effect parameter being zero was smaller than the cut-off value of 0.025. The oligonucleotide microarray data have been deposited in NCBI's Gene Expression Omnibus http://www.ncbi.nlm.nih.gov/geo following the MIAME guidelines and are accessible through GEO series accession number GSE24205.

### Mutation screening and genotyping

Mutation screening of the coding regions of selected genes and the genotyping of the P.Met1? mutation were performed by sequencing. Genomic DNA was extracted from peripheral blood samples using a commercially available kit (Puregene, Gentra Systems, Minneapolis, MN, USA). For sequencing analysis, PCR products were purified in 96-format Acro Prep Filter Plates (Pall Life Sciences, Ann Arbor, MI, USA) using the Perfect Vac Manifold vacuum machine (Eppendorf AG, Hamburg, Germany). Sequencing was performed according to the manufacturer's instructions using a BigDye Terminator v.3.1 Cycle Sequencing Kit and an automated ABI PRISM 3130xl Genetic Analyzer (Applied Biosystems, Foster City, CA, USA). Sequence analysis was done with Sequencher 4.2.2 software (Gene Codes Corporation, Ann Arbor, MI, USA).

### MicroRNA array protocol

MicroRNA expression levels in lymphoblastoid cell lines were detected using an Agilent Human miRNA V2 Oligo Microarray Kit (Agilent Technologies). First, 100 ng of total RNA was used as a starting material, and miRNAs were labeled using the Agilent miRNA Labeling Kit. Labeled RNA was hybridized to Agilent miRNA arrays with eight identical arrays per slide, with each array containing probes directed against 723 human and 76 human viral miRNAs. Slides were scanned (Agilent microarray scanner) after hybridization and data was extracted using Feature Extraction software, version 9.5.1. (Agilent Technologies). For data analysis, low quality samples and non-expressed miRNAs were first removed ending up with 29 individuals from 9 families and with 333 miRNAs. Inside every family the directional distance of healthy individuals from patients was calculated. The distance used here was based on Kendal's tau (distance = (1-tau)/2) and the distance between clusters was computed using Ward's method. By decomposing Kendal's tau into each miRNA's contribution the distance induced by every miRNA can be quantified separately. The direction of this distance inside every family is marked positive if the average rank miRNA expression for patients is higher than for healthy individuals and negative if the average rank miRNA expression for healthy individuals is higher than for patients. The overall directional distance for every miRNA is obtained by summing up these directional distances over all families. Then the permutation p-value was computed by permuting healthy individuals and patients randomly inside every family, computing the overall directional distance, repeating this many times and the final permutation p-value is the proportion of these permuted distances higher or lower than the original distance. The micro-RNA microarray data have been deposited in NCBI's Gene Expression Omnibus http://www.ncbi.nlm.nih.gov/geo following the MIAME guidelines and are accessible through GEO series accession number GSE24205.

### MicroRNA target detection

The miRanda algorithm [19] was used for finding genomic targets for miRNAs. For each miRNA, target genes were selected on the basis of three properties: sequence complementarity using a position-weighted local alignment algorithm, free energies of RNA-RNA duplexes, and conservation of target sites in related genomes. All of the human miRNA sequences were downloaded from the Sanger Institute miRBase [20], and they were aligned with the genomic sequences of variant sites from the sequenced genes. The aim was to identify miRNA target sites that either appear or disappear due to variants. Based on the total score value given to every wild type and mutant sequence combination by the miRanda algorithm, the difference between values was calculated. The top 5% of the highest differences were selected for further analysis.

### miRNA expression validation

Expression of miRNAs in the lymphoblastoid cell lines was validated by the specific TaqMan MicroRNA assays according to the manufacturer's instructions (Applied Biosystems). TaqMan microRNA assays were performed using Bio-Rad Laboratories' CFX384 real-time PCR detection system with the Bio-Rad C1000 thermal cycler (Bio-Rad Laboratories, Inc., US).

### Statistical analyses

Distribution of the genotypes and alleles, the odds ratio (OR), and the 95% confidence interval (CI) were calculated with the SPSS statistical software package, version 15.0 (SPSS, Chicago, IL). Associations with demographic, clinical, or pathological features of the disease (age at diagnosis, PSA value at diagnosis, T-stage, WHO grade, and Gleason score) were tested among unselected PC and HPC cases using R software http://www.r-project.org.

## Results

In order to identify genes containing inactivating mutations in the Xq27-q28 region, an NMD microarray analysis with Agilent 44 K Whole Human Genome oligonucleotide microarrays was performed in the families showing the strongest linkage to *HPCX1*. The candidate genes (n = 17) for subsequent sequence analysis were selected according to the microarray analysis (*RBMX*, *CSAG2*, *RAP2C*, *SOX3*, *MBNL3*, *ZNF75*, *MAGEC1*, *MAGEA1*, *MAGEA11*, *MAGEC3*, *MAGED1*, *U66046*, *SSR4*, *VBP1*, *LDOC1*, *TKTL1*, *CD40LG*) (Table 2). All identified stress response genes were excluded [21]. No truncating mutations were detected, but a total of 34 changes were found by direct sequencing. Eight of the changes were missense variants, six were silent changes, and twenty of the variants took place in introns, 5'UTR, or 3'UTR regions. Twenty of the changes were novel and not found in any genomic databases. A summary of the identified variants is presented in Table 3.

**Table 2:** Genes selected for resequencing based on NMD oligoarray analysis

Gene ID	Gene name	Cytogenetic band	Genomic location (strand)	Selection criteria
*RBMX*	RNA binding motif protein, X-linked	Xq26.3	135 951 351-135 962 939 bp (-)	p < 0.05
*CSAG2*	*Homo sapiens *CSAG family, member 2	Xq28	151 922 445-151 928 738 bp (+)	p < 0.05
*RAP2C*	*Homo sapiens *RAP2C, member of RAS	Xq25	131 337 053-131 353 471 bp (-)	p < 0.05, fold change > 1.5
*SOX3*	SRY (sex determining region Y)-box 3	Xq27.1	139 585 152-139 587 225 bp (-)	p < 0.05
*MBNL3*	Muscleblind-like 3, (*Drosophila*)	Xq26.2	131 506 029-131 623 996 bp (-)	p < 0.05
*ZNF75*	Zinc finger protein 75	Xq26.3	134 382 867-134 478 012 bp (-)	p < 0.05
*MAGEC1*	Melanoma antigen family C, 1	Xq26	140 991 680-140 997 183 bp (+)	p < 0.05
*MAGEA1*	Melanoma antigen family A, 1	Xq28	152 481 522-152 486 116 bp (-)	p < 0.05, fold change > 1.5
*MAGEA11*	Melanoma antigen family A, 11	Xq28	148 769 894-148,798,928 bp (+)	location
*MAGEC3*	Melanoma antigen family C, 3	Xq27.2	140 926 102-140 985 618 bp (+)	location
*MAGED1*	Melanoma antigen family D, 1	Xp11.23	51 546 103-51 645 453 bp (+)	p < 0.05
*U66046*	hypothetical protein FLJ44451	Xq28	148 615 451-148 616 127 bp (-)	p < 0.05
*SSR4*	signal sequence receptor, delta	Xq28	153 058 971-153 063 960 bp (+)	p < 0.05
*VBP1*	von Hippel-Lindau binding protein 1	Xq28	154 444 550-154 468 098 bp (+)	p < 0.05
*LDOC1*	leucine zipper, down-regulated in cancer, 1	Xq27	140 269 930-140 271 310 bp (-)	p < 0.05
*TKTL1*	transketolase-like 1	Xq28	153 524 024-153 558 700 bp (+)	p < 0.05
*CD40LG*	CD40 ligand	Xq26	135 730 336-135 742 549 bp (+)	p < 0.05

**Table 3:** Summary of variants found in the *HPCX1 *region from Finnish HPC families

Gene	Variation*	Amino acid change	dbSNP
*CD40LG*	c.148T > C	p.Leu50Ser	rs1126535
*LDOC1*	c.-62C > G	-	-
*MAGEA1*	c.-2924G > T	-	-
	c.-264, G > A	-	-
	c.813C > T	p.Leu271	rs2233045
	c.*17A > G	-	-
	c.*477C > T	-	-
*MAGEA11*	c.96+38A > G	-	-
	c.193-64T > C	-	-
	c.266+10C > T	-	-
	c.1077 C > T	p.Leu359	-
	c.*234T > C	-	-
*MAGEC1*	c.-2051G > A	-	-
	c.-2008T > C	-	rs1003682
	c.2T > C	P.Met1?	-
	c.5-44T > C	-	-
	c.74G > A	p.Cys25Tyr	rs176036
	c.452C > T	p.Thr151Ile	rs176037
	c.1327C > G	p.Leu443Val	rs62611966
	c.1401C > G	p.His467Gln	rs176048
	c.2125C > T	p.His709Tyr	rs56256227
	c.3174G > A	p.Glu1058	rs12845617
	c.*53C > T	-	rs41300301
*MAGEC3*	c.-189C > T	-	-
	c.259-66G > A	-	-
	c.259-15T > C	-	-
	c.880C > G	p.Leu294Val	-
	c.958C > T	p.Leu320	rs176025
*MBNL3*	c.923-11G > A	-	-
*RBMX*	c.-1C > A	-	rs2011584
*SUHW3*	c.1851A > C	p.Gly617	rs209238
	c.2161+4C > T	-	-
	c.2162-41T > C	-	-
*ZNF75*	c.1434 G > A	p.Thr478	rs1129093

*Numbering is according to the cDNA starting at the A in the start codon.

The most interesting variation selected for follow-up was the *MAGEC1 *p.Met1? (c.2T > C; Met > Thr) start codon mutation found in family 311. The frequency of that mutation was determined by sequencing among patients with unselected PC or HPC and in the control groups. The carrier frequencies for p.Met1? were 2.45%, 1.72%, 0.92%, 0.53%, 1.21%, and 0.65% in the probands with HPC, unselected PC cases, male population controls, BPH controls, PSA controls, and female population controls, respectively. The frequency of P.Met1? was found to be in Hardy-Weinberg equilibrium in controls. A statistically significant difference was observed in the carrier frequencies of the P.Met1? variant between the sample groups, and an association was seen between the variant and unselected PC and HPC (see Table 4). The association was strongest when male and female blood donors and BPH patients were used as a control group (OR = 2.35, 95% CI = 1.10-5.02 for unselected PC; OR = 3.38, 95% CI = 1.10-10.40 for HPC). The association between the frequency of the variant and the disease phenotype, including tumor WHO grade, Gleason score, T-stage, age at diagnosis, and PSA value at diagnosis, was also analyzed among the unselected PC cases as no complete data were available for the HPC cases. No significant associations were found from these studies (data not shown). Additional samples from three prostate cancer families carrying the p.Met1? variant were also analyzed, but cosegregation of the p.Met1? was incomplete (data not shown) which is quite expected for a low penetrance gene.

**Table 4:** Association of the *MAGEC1 *P.Met1? variant with unselected PC or HPC

Sample group	Carrier frequency	OR (95% CI)	P
Male population controls	7/757 (0.92%)	1.00	
Female population controls	5/764 (0.65%)	1.00	
Patients with BPH	2/375 (0.53%)	1.00	
PSA controls	9/746 (1.21%)	1.00	
Patients with unselected PC	13/757 (1.72%)	1.99 (1.00-3.95)*	0.04*
		2.35 (1.10-5.02)**	0.02**
Patients with HPC	4/163 (2.45%)	2.86 (0.98-8.38)*	0.04*
		3.38 (1.10-10.40)**	0.02**

*Male and female blood donors, BPH patients, and PSA controls used as a control group.

**Male and female blood donors and BPH patients used as a control group.

**Figure 1:**
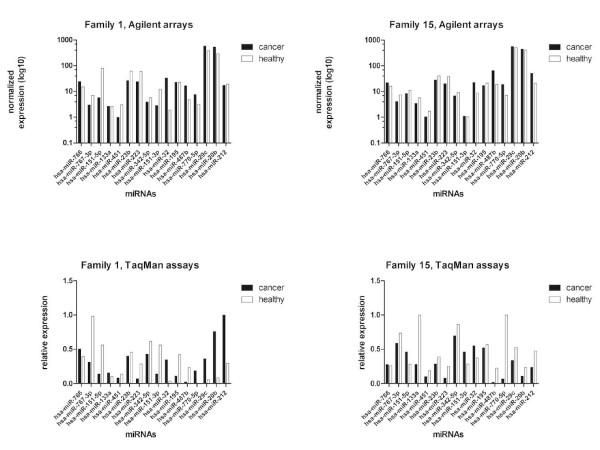
**Validation of miRNA expressions in families 1 and 15**. In the upper row the normalized expressions of sixteen miRNAs are presented (black bars indicate cancer patients and white bars indicate healthy brothers). In the lower row the corresponding expression values measured with specific TaqMan miRNA assays are displayed.

**Figure 2:**
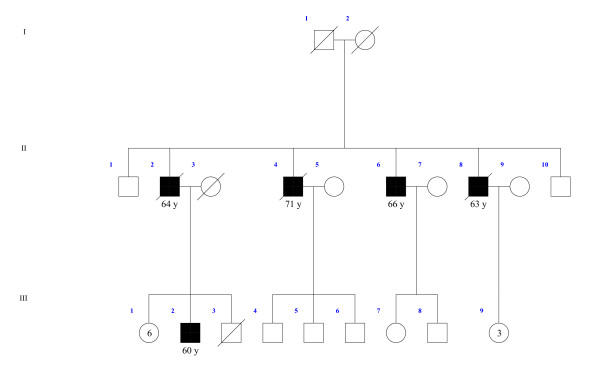
**Pedigree of *HPCX1 *linked family 1**. Black square denotes persons with prostate cancer, black square and circle with white surroundings signifies patient with another type of cancer. Age at diagnosis for prostate cancer patients (in years) is indicated below the symbol. The pedigrees have been altered to protect anonymity.

**Figure 3:**
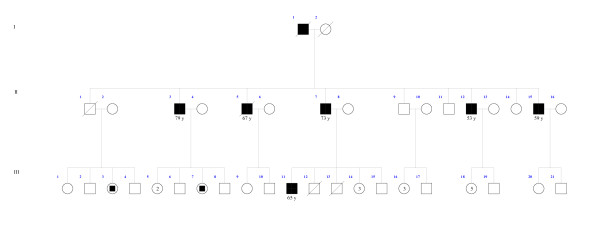
**Pedigree of *HPCX1 *linked family 15**. Symbols are similar as in Figure 2.

MicroRNA expression levels in lymphoblastoid cell lines were determined with Agilent Human miRNA arrays. The most differentially expressed miRNAs when testing against both alternatives (miRNA expression is either up- or downregulated in case vs. controls) are presented in Table 5 and 6 together with information about genomic and intronic/intergenic location. The miRanda algorithm produced 1211 different variant-miRNA combinations with a total score value above the cut-off value. From the 29 differentially expressed miRNAs between patients and healthy individuals twelve miRNAs for validation were selected based on that they supposedly had a target site in *MAGEC1 *gene (Table 5 and 6). In addition, miRNAs located in X chromosome were selected for validation. Validation of these selected miRNA expressions in cell lines was based on specific TaqMan MicroRNA Assays (Tables 5 and 6, Figures 1, 2 and 3).

**Table 5:** Differentially expressed miRNAs between patients and healthy individuals: Testing against alternative that a miRNA is upregulated in patients

Mature miRNA^1^	p-value	Pre-miR miRBase ID	Location	Possible target site in *MAGEC1*	Validation (TaqMan)
hsa-miR-296-5p	0.0049	MI0000747	chr 20: intronic (AL136532)		
**hsa-miR-766**	**0.0157**	**MI0003836**	**chr X: intronic (*SEPT6*)**		**+ (in families 1,15,232,362)**
**hsa-miR-767-3p**	**0.0189**	**MI0003763**	**chr X: intronic (*GABRA3*)**		**+ (in families** **1, 15)**
**hsa-miR-151-5p**	**0.0312**	**MI0000809**	**chr 8: intronic (*PTK2*)**	**c.5-44T > C** c.*53C > T	**+ (in family 1)**
**hsa-miR-133a**	**0.0316**	**133a-1: MI0000450**	**chr 18: intronic (*MIB1*)**	**c.74G > A**	**+ (in family 15)**
		**133a-2: MI0000451**	**chr 20: intronic (*C20orf166*)**		
**hsa-miR-451**	**0.0319**	**MI0001729**	**chr 17: intergenic**	**c.1327C > G**	**+ (in family 1)**
**hsa-miR-23b**	**0.0393**	**MI0000439**	**chr 9: intronic (*C9orf3*)**	**c.452C > T** **c.1327C > G**	**+ (in families 1, 15, 413)**
**hsa-miR-223**	**0.0404**	**MI0000300**	**chr X: intergenic**		**+ (in families 1, 15, 362, 413)**
hsa-miR-146a*	0.0404	MI0000477	chr 5: intergenic		
**hsa-miR-342-5p**	**0.0410**	**MI0000805**	**chr 14: intronic (*EVL*)**	**c.452C > T**	**+ (in families 248, 292, 402, 408, 413)**
hsa-miR-183*	0.0433	MI0000273	chr 7: intergenic		
**hsa-miR-151-3p**	**0.0438**	**MI0000809**	**chr 8: intronic (*PTK2*)**	**c.5-44T > C** c.*53C > T	**+ (in families 1, 402)**
hsa-miR-129-3p	0.0465	MI0000473	chr 11: intergenic		
hsa-miR-129*	0.0477	MI0000252	chr 7: intergenic		

^1^miRNAs selected for validation are represented in bold

**Table 6:** Differentially expressed miRNAs between patients and healthy individuals: Testing against alternative that a miRNA is downregulated in patients

Mature miRNA^1^	p-value	Pre-miR miRBase ID	Location	Possible target site in *MAGEC1*	Validation (TaqMan)
**hsa-miR-32**	**0.0001**	**MI0000090**	**chr 9: intronic (*C9orf5*)**	c.*53C > T	**+ (in families 1, 248)**
hsa-miR-30a	0.0102	MI0000088	chr 6: intronic (*C6orf155*)		
hsa-miR-33a	0.0184	MI0000091	chr 22: intronic (*SREBF2*)		
hsa-miR-345	0.0210	MI0000825	chr 14: intergenic		
**hsa-miR-195**	**0.0245**	**MI0000489**	**chr 17: intronic (*AC027763*)**	c.*53C > T	**+ (in families 292, 362, 408)**
**hsa-miR-487b**	**0.0279**	**MI0003530**	**chr 14: intergenic**	c.*53C > T	**+ (in families 248, 292)**
hsa-miR-370	0.0292	MI0000778	chr 14: intergenic		
**hsa-miR-770-5p**	**0.0305**	**MI0005118**	**chr 14: intronic (*MEG3*)**	**c.3174G > A**	**-**
**hsa-miR-29c**	**0.0330**	**MI0000735**	**chr 1: intergenic**	**c.1327C > G** **c.2125C > T**	**+ (in families 1, 248, 362)**
hsa-miR-34c-3p	0.0351	MI0000743	chr 11: intergenic		
hsa-miR-148a	0.0426	MI0000253	chr 7: intergenic		
**hsa-miR-20b**	**0.0431**	**MI0001519**	**chr X: intergenic**		**+ (in family 1)**
hsa-miR-29b	0.0478	29b-1: MI0000105	chr 7: intronic (*AC016831*)		
		29b-2: MI0000107	chr 1: intergenic		
**hsa-miR-212**	**0.0482**	**MI0000288**	**chr 17: intergenic**	**c.3174G > A**	**-**
hsa-miR-7	0.0490	7-1: MI0000263	chr 9: intronic (*HNRNPK*)		
		7-2: MI0000264	chr 15: intergenic		
		7-3: MI0000265	chr 19: intronic (*C19orf30*)		

^1^miRNAs selected for validation are represented in bold

## Discussion

The recognition that PC clusters within families has led to the collection of HPC families with the goal of localizing and identifying PC susceptibility genes. Initially, the PC susceptibility locus, *HPCX1*, was mapped to the X chromosome by using a set of high-risk PC families from the United States, Finland, and Sweden. The effect of *HPCX1 *was proven to be the most important in the Finnish population, where a specific haplotype in the region was also identified [5,6]. Further, X-chromosomal inheritance coheres with the results of our segregation analysis of multifactorial recessive inheritance as the only model in the Finnish population [22]. In this study, a recently developed NMD microarray technology was utilized for the analysis of the *HPCX1 *region with brother pairs from *HPCX1 *linked families. Use of the unaffected brothers as controls might be problematic given the late age of prostate cancer diagnosis, but the unaffected brothers were chosen to be the oldest possible from every family with no history of PC. Previously, the manipulation of NMD together with expression array analysis has proven to be a powerful tool for detecting novel gene mutations in cancer cells. Huusko et al. [23] successfully identified *EPHB2 *gene mutations in PC. Since then, mutations in melanoma cell lines [24], colon cancer cell lines [25], and PC cell lines [26] have been identified by inhibition of NMD.

Sequencing of 17 genes from the *HPCX1 *region did not reveal any truncating nonsense mutations. The most interesting variation for follow-up was in the start codon (p.Met1?) of the *MAGEC1 *gene. An association was later seen between the p.Met1? variant and unselected PC and HPC. Interestingly, the association was strongest when "supernormal" PSA controls were excluded from the control group. The PSA controls consisted of men with PSA levels < 1.0 ng/ml and a mean age of 67.5 years, which is lower than the mean age of BPH patients (73 years). As *HPCX1 *is suggested to be a late-onset disease in the Finnish population [5], as well as in some other populations [27], it is possible that the younger PSA control group is actually more saturated with the p.Met1? individuals who are at risk of developing late-onset HPCX. *MAGEC1 *is a member of the melanoma antigen gene (MAGE) family [28]. The proteins of this family are tumor-specific antigens that can be recognized by autologous cytolytic T lymphocytes. *MAGEC1 *is composed of four exons and encodes a protein of 1142 amino acids. It is approximately 800 residues longer than other MAGE proteins due to the insertion of a large number of short repetitive sequences in front of the MAGE-homologous sequence. *MAGEC1 *is expressed in a significant proportion of tumors of various histological types, but is silent in normal tissues, excluding the testis. Alternative start codons, mainly GTG and TTG, are used in prokaryotes and, very rarely, in higher organisms. One example is the vitamin-D receptor (*VDR*) gene start codon polymorphism, where a T/C polymorphism in the first of the two potential start (ATG) codons results in two alleles that can be distinguished by RFLP using the endonuclease FokI [29]. The biological function of the identified *MAGEC1 *start codon variant in this case is difficult to assess since we were not able to investigate the expression of *MAGEC1 *in individuals carrying the mutated allele in the absence of other tissue material. Considering the conserved structure and similar functions of MAGE proteins, it might be possible that other members of the gene family can partly compensate for the functions of *MAGEC1 *if the start codon mutation totally blocks the translation of the gene.

In this NMD array analysis, the false positive rate was evidently high since no truncating mutations were found. Emetine treatment followed by actinomycin D treatment was used, but it has been observed that treatment with actinomycin D after emetine incubation does not have a significant effect on treated cells, suggesting that the combination of the drugs is not the best possible method for this type of study [30]. A novel improvement to the NMD protocol includes a combination of emetine and caffeine treatment [25], which leads to a more efficient identification of false positives produced by cell stress. On the other hand, we might have missed genes that actually carry truncating mutations. If the genes were mutated both in the seemingly healthy males with a normal clinical phenotype and their already affected siblings, they would have had the same profile as the stress response genes and would have, therefore, been excluded. In addition, relevant variants might have been missed by being limited to use lymphoblastoid cell lines, as they may not resemble the whole set of active genes in prostate tissue. Although there is substantial amount of evidence that lymphoblastoid cells encompasses a variety of metabolic pathways that are specific to individuals where the cells originated, making these cell lines suitable for molecular and functional studies [31].

Despite its association with PC, the segregation of the P.Met1? variant in studied families was incomplete. This finding can also reflect the high phenocopy rate of the disease, and as a consequence, even the most extreme families may have a few sporadic cases. Currently, there are no clinical or molecular tools to distinguish "true" HPC cases from sporadic ones. Therefore, the incomplete segregation does not necessarily diminish the importance of p.Met1? as a *HPC*X*1 *risk variant.

The number of publications reporting differential miRNA expression in prostate cancer is constantly growing. Analyses have been performed for example in clinical prostate specimens [32], and in PC cell lines, xenografts, BPH vs. PC samples [33]. Most recently, miRNA expression profiles of androgen-responsive and castrate-resistant PC cell lines were compared [34]. In all of these studies, a set of 20-30 miRNAs unequivocally differentiated the PC samples from the normal samples and non-malignant precursor lesions, and specific miRNAs including miR-125b, miR-145, and let-7c were repeatedly detected differentially expressed in different studies.

In the current study, we hypothesized that miRNA profiling could be used as a tool for discovering variants from non-protein coding regions which could explain the "dark" inheritance behind HPCX. Altered miRNA expression in patients lymphoblastoid cells could lead to the identification of germline variants in promoter or other regulatory regions of protein coding genes since considerable amount of miRNA expression is correlated to host and target gene expression [35]. Possible explanation of differences in miRNA levels could be their role in tumor development and initiation. Already, several studies have shown that miRNAs in serum can be considered as biomarkers [36,37] and serum miRNAs are mainly derived from circular blood cells [38], the same way as lymphoblastoid cells. We were able to identify 29 significantly differently expressed miRNAs between patients and their healthy brothers. Validation of the expressions of the selected 16 miRNAs with TaqMan assays were successful in 14/16 cases, including all four miRNAs located in the X chromosome.

## Conclusions

In conclusion, we suggest a role for *MAGEC1 *in genetic PC susceptibility, especially in the *HPCX1*-linked form of the disease. The start codon missense variation in the *MAGEC1 *gene showed a borderline association between the variant and both HPC and unselected PC, and therefore additional research is warranted. In addition, the role of certain miRNAs needs further study, especially since *MAGEC1 *was predicted to be one of their targets.

## List of abbreviations used

HPC: hereditary prostate cancer; NMD: nonsense-mediated mRNA decay; PC: prostate cancer; TSG: tumor suppressor gene; GINI: gene identification by NMD inhibition; BPH: benign prostate hyperplasia; PSA: prostate specific antigen

## Competing interests

The authors declare that they have no competing interests.

## Authors' contributions

HM participated in the design of the study, carried out the array and sequencing studies and drafted the manuscript. MS and JI carried out the array data analysis and statistical analyses and revised the manuscript. TI participated in the selection of patients, design of the study and revised the manuscript. MV performed the miRanda studies and revised the manuscript. HO participated in the design of the study and contributed to the array data analysis. TT is the clinical contributor. TW contributed to study design and coordination and revised the manuscript. JS participated in study design, interpreted the results and critically revised the manuscript. All the authors have read and approved the final manuscript.

## Pre-publication history

The pre-publication history for this paper can be accessed here:

http://www.biomedcentral.com/1471-2407/11/327/prepub
